# Mechanisms responsible for increased circulating levels of galectin-3 in cardiomyopathy and heart failure

**DOI:** 10.1038/s41598-018-26115-y

**Published:** 2018-05-29

**Authors:** My-Nhan Nguyen, Yidan Su, Donna Vizi, Lu Fang, Andris H. Ellims, Wei-Bo Zhao, Helen Kiriazis, Xiao-Ming Gao, Junichi Sadoshima, Andrew J. Taylor, Julie R. McMullen, Anthony M. Dart, David M. Kaye, Xiao-Jun Du

**Affiliations:** 1 Baker Heart and Diabetes Institute, Melbourne, Australia; 2 Central Clinical School, Monash University, Melbourne, Australia; 3 Department of Cardiovascular Medicine, the Alfred Hospital, Melbourne, Australia; 4 Department of Cardiology, Southwest Hospital, Third Military Medical University, Chongqing, China; 5 Department of Cell Biology and Molecular Medicine Rutgers, New Jersey Medical School, New Jersey, USA

## Abstract

Galectin-3 is a biomarker of heart disease. However, it remains unknown whether increase in galectin-3 levels is dependent on aetiology or disease-associated conditions and whether diseased heart releases galectin-3 into the circulation. We explored these questions in mouse models of heart disease and in patients with cardiomyopathy. All mouse models (dilated cardiomyopathy, DCM; fibrotic cardiomyopathy, ischemia-reperfusion, I/R; treatment with β-adrenergic agonist isoproterenol) showed multi-fold increases in cardiac galectin-3 expression and preserved renal function. In mice with fibrotic cardiomyopathy, I/R or isoproterenol treatment, plasma galectin-3 levels and density of cardiac inflammatory cells were elevated. These models also exhibited parallel changes in cardiac and plasma galectin-3 levels and presence of trans-cardiac galectin-3 gradient, indicating cardiac release of galectin-3. DCM mice showed no change in circulating galectin-3 levels nor trans-cardiac galectin-3 gradient or myocardial inflammatory infiltration despite a 50-fold increase in cardiac galectin-3 content. In patients with hypertrophic cardiomyopathy or DCM, plasma galectin-3 increased only in those with renal dysfunction and a trans-cardiac galectin-3 gradient was not present. Collectively, this study documents the aetiology-dependency and diverse mechanisms of increment in circulating galectin-3 levels. Our findings highlight cardiac inflammation and enhanced β-adrenoceptor activation in mediating elevated galectin-3 levels via cardiac release in the mechanism.

## Introduction

Galectin-3 (Gal-3) is a β-galactoside-binding lectin that binds to glycoproteins thereby regulating their activities^1^. Clinical studies have shown that high circulating Gal-3 levels are indicative of severity of heart diseases or associated with increased risk of major adverse cardiovascular events including heart failure (HF)^2^, arrhythmias^3–5^, arterial stiffening^6^, re-hospitalization post-HF discharge^7^, diastolic dysfunction^8^, severity of atrial fibrosis^5,9^ or mortality^10,11^. However, a lack of association of Gal-3 levels and disease severity has also been reported in some studies^12–15^.

The mechanism(s) responsible for increased blood levels of Gal-3 remains incompletely defined. While increased cardiac Gal-3 expression was observed in human cardiac biopsies^5,9,16,17^, cardiac release of Gal-3 is not evident in patients with atrial fibrillation or severe HF indicated by the absence of a trans-cardiac Gal-3 gradient^18,19^. A positive correlation between blood and myocardial levels of Gal-3 was reported in one study^20^, but not in another study^16^. Clinical studies have consistently reported a strong and negative correlation between circulating Gal-3 levels and estimated glomerular filtration rate (eGFR), indicating that renal dysfunction is a determinant of blood Gal-3 levels^13,21–23^. Indeed, Gal-3 levels are markedly elevated in patients with end-stage renal failure^24^.

There is an increasing appreciation of the presence of inflammation contributing to HF^25^. In patients with dilated (DCM) or inflammatory cardiomyopathy, cardiac expression of Gal-3 correlated with inflammatory cell density^16^. In this context, myocardial infarction (MI) is known to trigger intense regional and systemic inflammation^26^, and in patients with acute MI, blood levels of inflammatory biomarkers including Gal-3 are increased^27^. Another hallmark of heart disease is activation of the sympatho-β-adrenergic system^28^. So far, there has been no study that has addressed whether activation of β-adrenoceptors (β-AR) affects circulating Gal-3 levels.

We have investigated factors responsible for increased circulating Gal-3 in cardiomyopathy by addressing the following questions: *First*, whether changes in circulating Gal-3 levels is aetiology-dependent; *Second*, whether the inflammatory state of heart disease is associated with increased circulating Gal-3; *Third*, whether activation of β-ARs alters cardiac and circulating Gal-3 levels; *Finally*, whether there is Gal-3 release from the heart into the circulation. We studied mouse models of cardiomyopathies and mice with ischemia/reperfusion (I/R) or treatment with the β-AR agonist isoproterenol. Two cohorts of patients with hypertrophic cardiomyopathy (HCM) or DCM and ischemic cardiomyopathy (ICM) were also studied.

## Results

### Blood and cardiac levels of Gal-3 in mouse models of cardiomyopathy or ischemia/reperfusion

**Figure 1:**
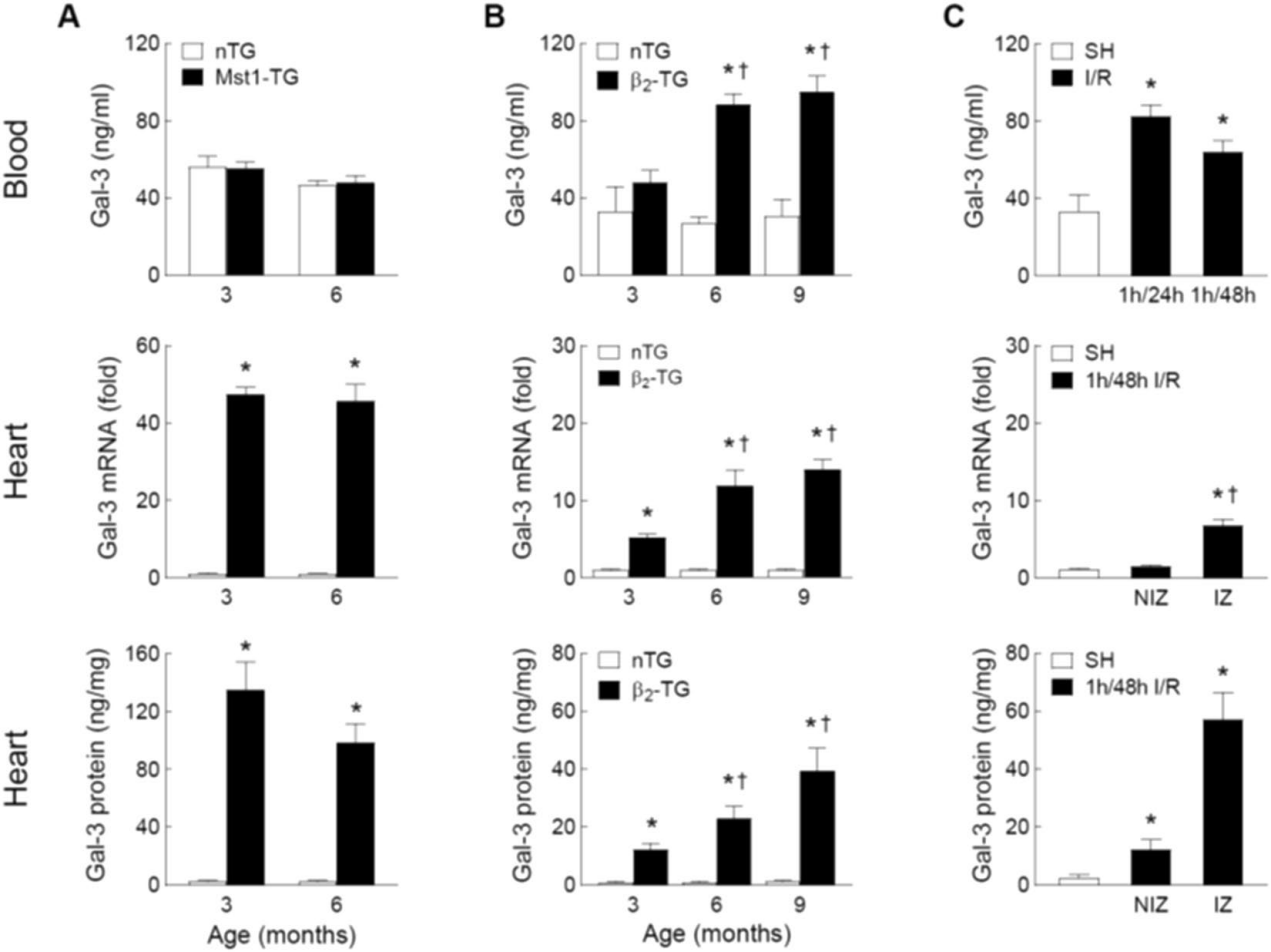
Plasma and myocardial levels of galectin-3 in the mouse models of cardiomyopathy and ischemia-reperfusion (I/R). Changes in plasma concentrations of Gal-3 and cardiac expression of Gal-3 at mRNA and protein levels in Mst1-TG and non-TG (nTG) mice at 3 and 6 months of age (n = 6∼8/group, **A**) in β_2_-TG (n = 5∼9/group) and nTG mice (n = 4∼6/group) at 3, 6 and 9 months of age (**B**) and in mice subjected to 1-hour ischemia followed by reperfusion of 24 or 48 hours (n = 5∼7/group) or sham-operation as control (SH, n = 5, **C**). In hearts from mice subjected to I/R (1/48 h, n = 5) or SH (n = 4), the infarct zone (IZ) was separated from the non-infarct zone (NIZ) and the assays were performed separately. Mean ± SEM. *P < 0.05 vs. nTG or SH, and ^†^*P* < 0.05 vs. 3-month-old β_2_-TG or NIZ.

In Mst1-TG mice aged at 3 and 6 months, blood levels of Gal-3 were comparable to that of nTG littermates (Fig. 1A) despite a 50-fold increase in cardiac Gal-3 expression at mRNA and protein levels (*P* < 0.05 vs. nTG, Fig. 1A). The β_2_-TG and nTG mice were studied at 3, 6 and 9 months, respectively. Relative to age-matched nTG levels, plasma Gal-3 concentrations were age-dependently elevated in β_2_-TG mice (Fig. 1B). In β_2_-TG mice, cardiac Gal-3 expression was age-dependently increased by 5∼20-fold at mRNA and protein levels (Fig. 1B).

For comparison with the cardiomyopathic models, I/R model was similarly studied. Mice with I/R (1/24 h or 1/48 h) showed a 2-fold increase in plasma Gal-3 levels (*P* < 0.05 vs. SH, Fig. 1C). In the ischemic myocardium (i.e. infarct zone, IZ), Gal-3 was upregulated by 7-fold at mRNA level and 30-fold at protein level (Fig. 1C). In the non-infarct zone (NIZ), Gal-3 increased by 5-fold at protein level whilst mRNA level were not significantly different versus SH group (Fig. 1C).

**Figure 2:**
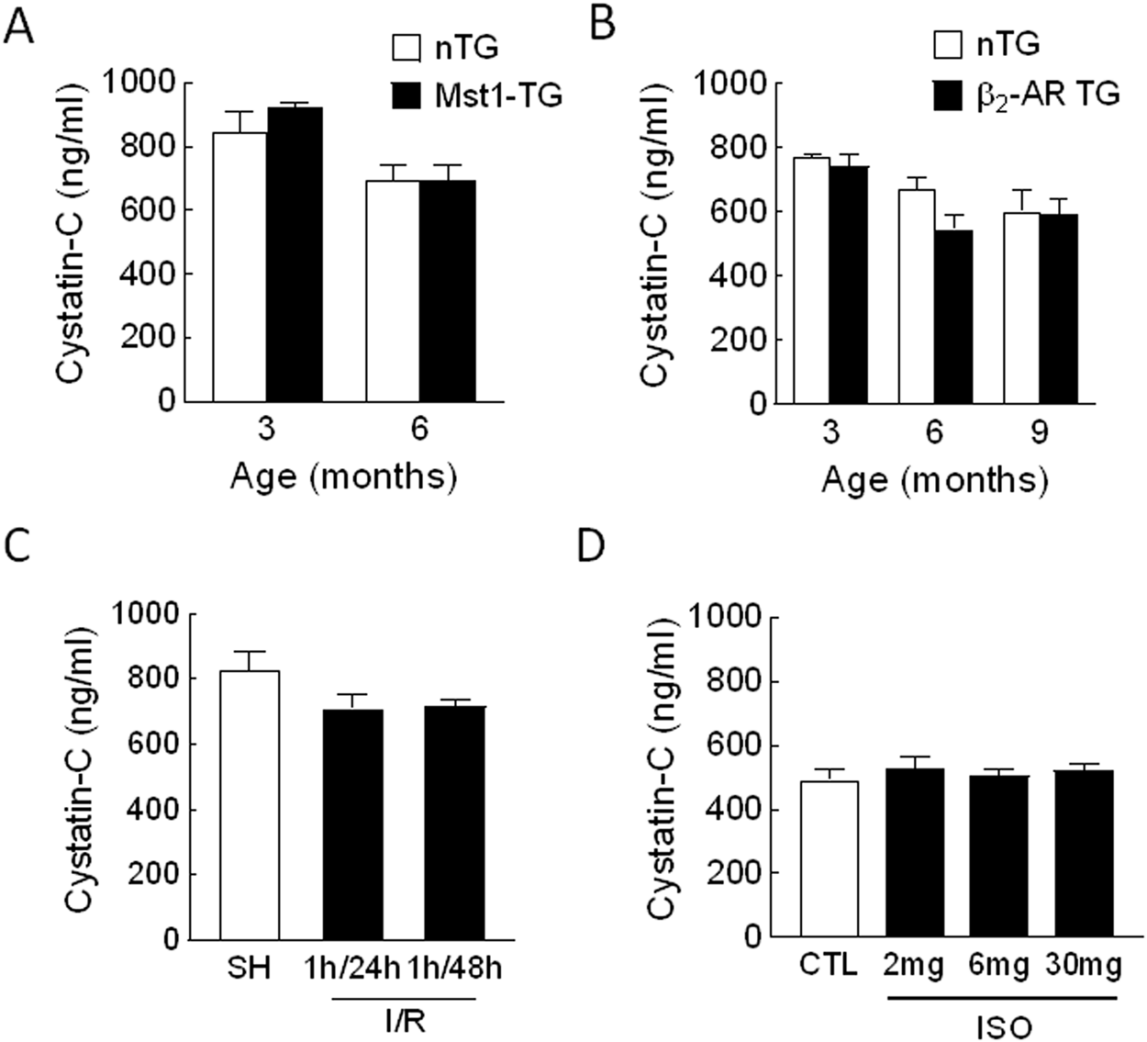
Plasma levels of the renal biomarker cyctatin-C in mouse models of heart disease. Mouse models consist of transgenic cardiomyopathy due to cardiac overexpression of Mst1 (**A**) or β_2_-AR (**B**) and mice subjected to ischemia/reperfusion (I/R, **C**) or treatment with isoproterenol (ISO, **D**). N = 5∼9 per group in all experiments.

In all three models, circulating levels of renal biomarker cystatin-C were comparable between diseased and control animals (Fig. 2A–C).

### Inflammatory parameters in mouse models of cardiomyopathy or ischemia/reperfusion

**Figure 3:**
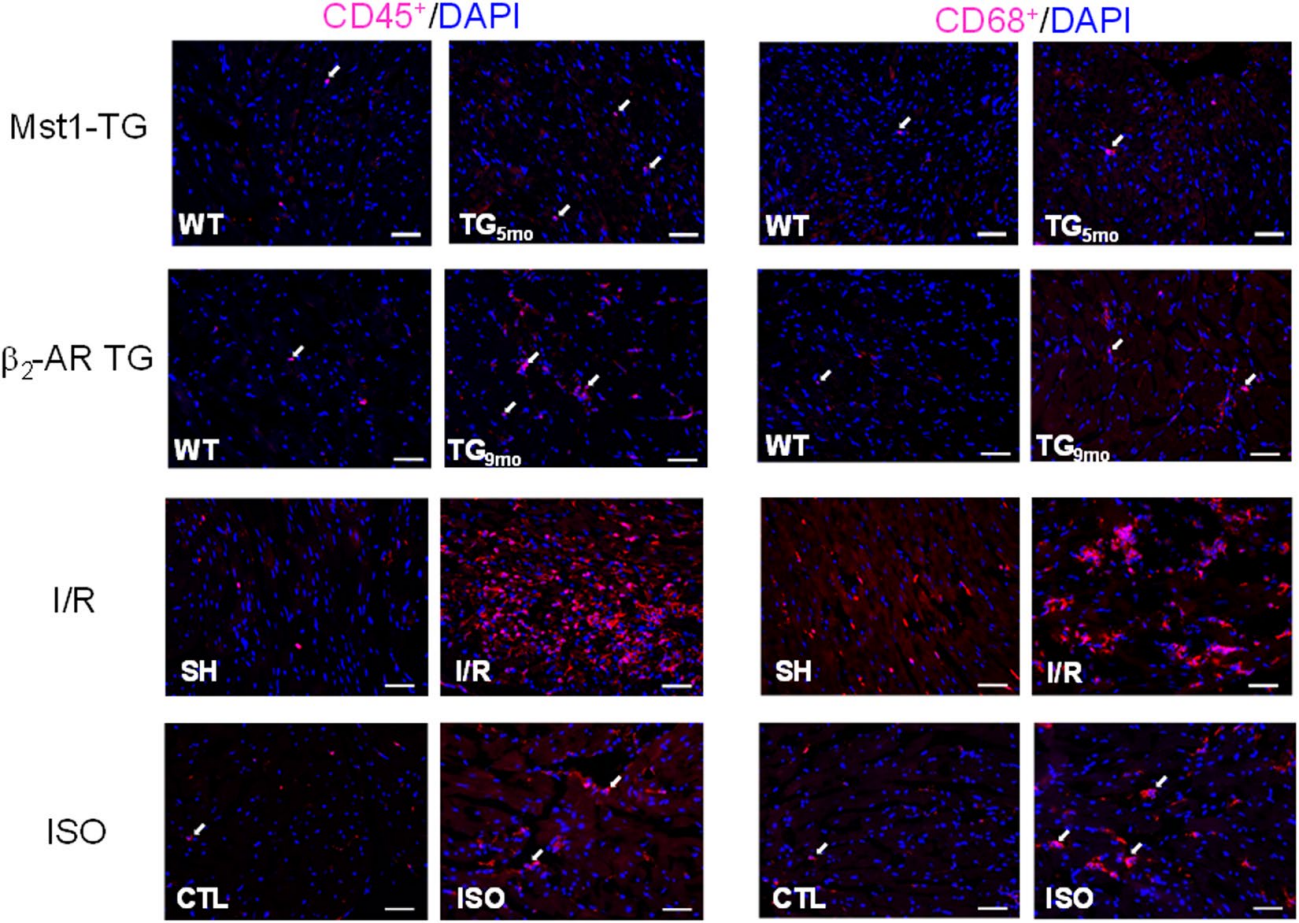
Representative histological images of CD45 and CD68 cells by immunohistochemistry of the left ventricles. Images were from of two transgenic mouse models of cardiomyopathy, mice with ischemia/reperfusion (I/R, 1/48 h) or treated with isoproterenol (ISO, 6 mg/kg for 48 hours). Arrows indicate positively stained cells. Bar = 50 μm.

The degree of inflammation was estimated by determining the density of leukocytes (CD45^+^) and macrophages (CD68^+^) in the myocardium and also by gene expression of Gal-3, MMP-9 and TNF-α in isolated circulating leukocytes. Figure 3 show representative images of immunohistochemical staining of CD45^+^ and CD68^+^ cells all animal models studied.

**Figure 4:**
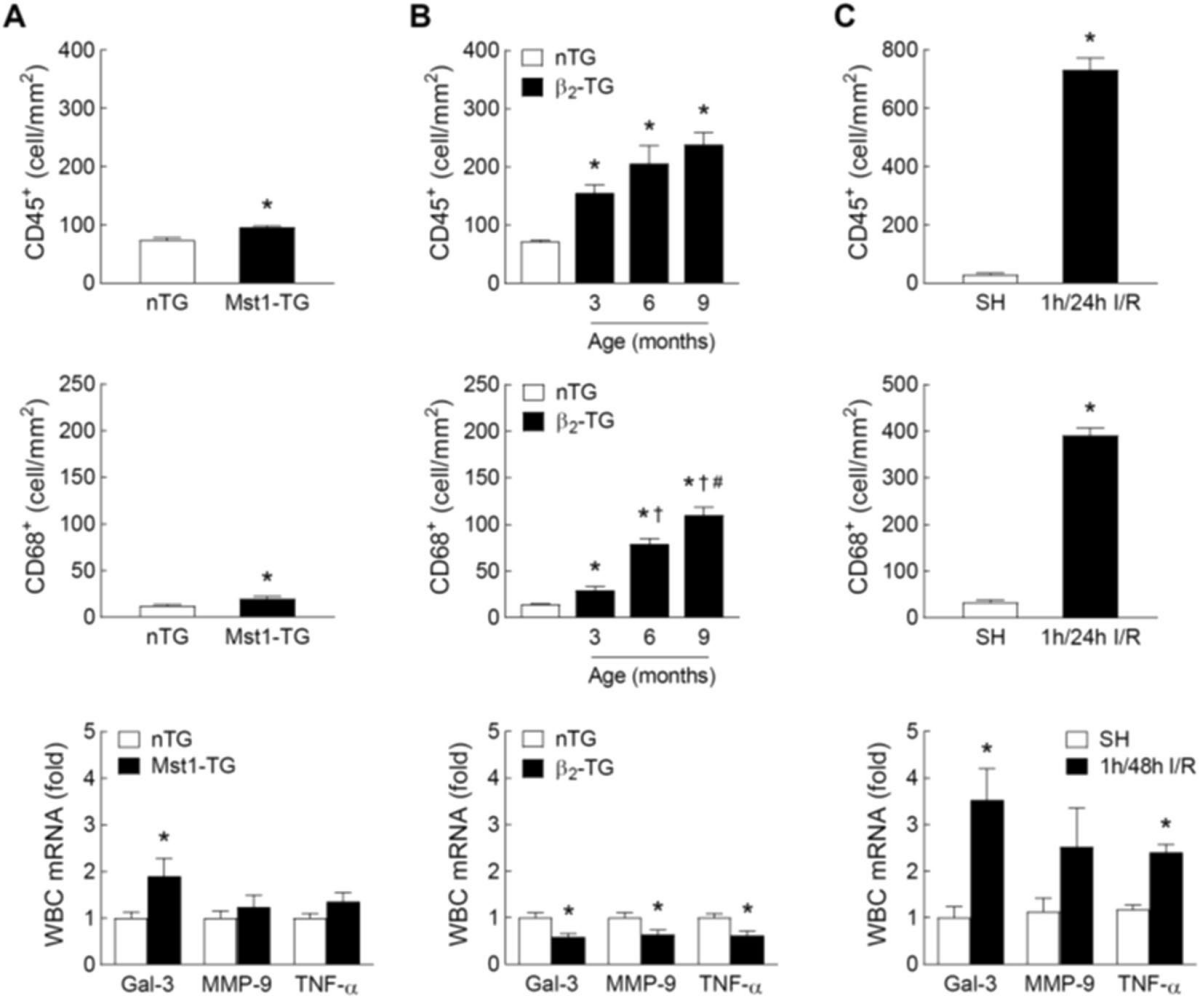
Inflammatory parameters of the myocardium and circulating leukocytes in mouse models of cardiomyopathy and ischemia-reperfusion (I/R). Cardiac and systemic inflammation was assessed by immunohistochemistry-based quantification of leukocytes (CD45^+^) or macrophages (CD68^+^) in the LV myocardium and by gene expression of Gal-3, matrix metalloproteinase-9 (MMP-9) and tumour necrosis factor-α (TNF-α) in circulating leukocytes. Results were from (**A**) Mst1-TG and nTG mice (n = 6/group for immunohistochemistry and n = 9/group for gene expression, 3–6 months of age). Data were combined from mice aged 3 and 6 months of age; (**B**) β_2_-TG and nTG mice aged at 3, 6 and 9 months (n = 5∼8 per age group for immunohistochemistry and n = 6∼12/group for gene expression of circulating leukocytes from 6-month-old mice); and (**C**) mice subjected to I/R (1/48 h) or sham-operation (SH) (n = 7/group for immunohistochemistry and n = 5∼8/group for gene expression). Mean ± SEM. *P < 0.05 vs. nTG or SH, ^†^*P* < 0.05 vs. 3-month-old β_2_-TG, ^#^*P* < 0.05 vs. 6-month-old β_2_-TG.

In Mst1-TG hearts, densities of CD45^+^ and CD68^+^ cells were marginally increased versus nTG controls (Fig. 4A). In circulating leukocytes of Mst1-TG, Gal-3 gene expression was increased but expression of MMP-9 and TNF-α was unchanged relative to nTG values (Fig. 4A). β_2_-TG hearts exhibited a significant and age-dependent increase in the density of CD45^+^ and CD68^+^ cells versus nTG hearts (Fig. 4B). In circulating leukocytes, gene expression of Gal-3, MMP-9 and TNF-α was 40% lower in β_2_-TG than nTG mice (Fig. 4B). The I/R model was characterised by a robust increase in the densities of CD45^+^ and CD68^+^ cells within the ischemic region (*P* < 0.05 vs. SH, Fig. 4C). Related to SH group, there was a 2∼3-fold upregulation in the expression of Gal-3 (*P* < 0.05), MMP-9 (*P* = 0.2) and TNF-α (*P* < 0.05) in circulating leukocytes from I/R mice (Fig. 4C).

### Upregulation of circulating and cardiac Gal-3 by β-AR activation with isoproterenol

**Figure 5:**
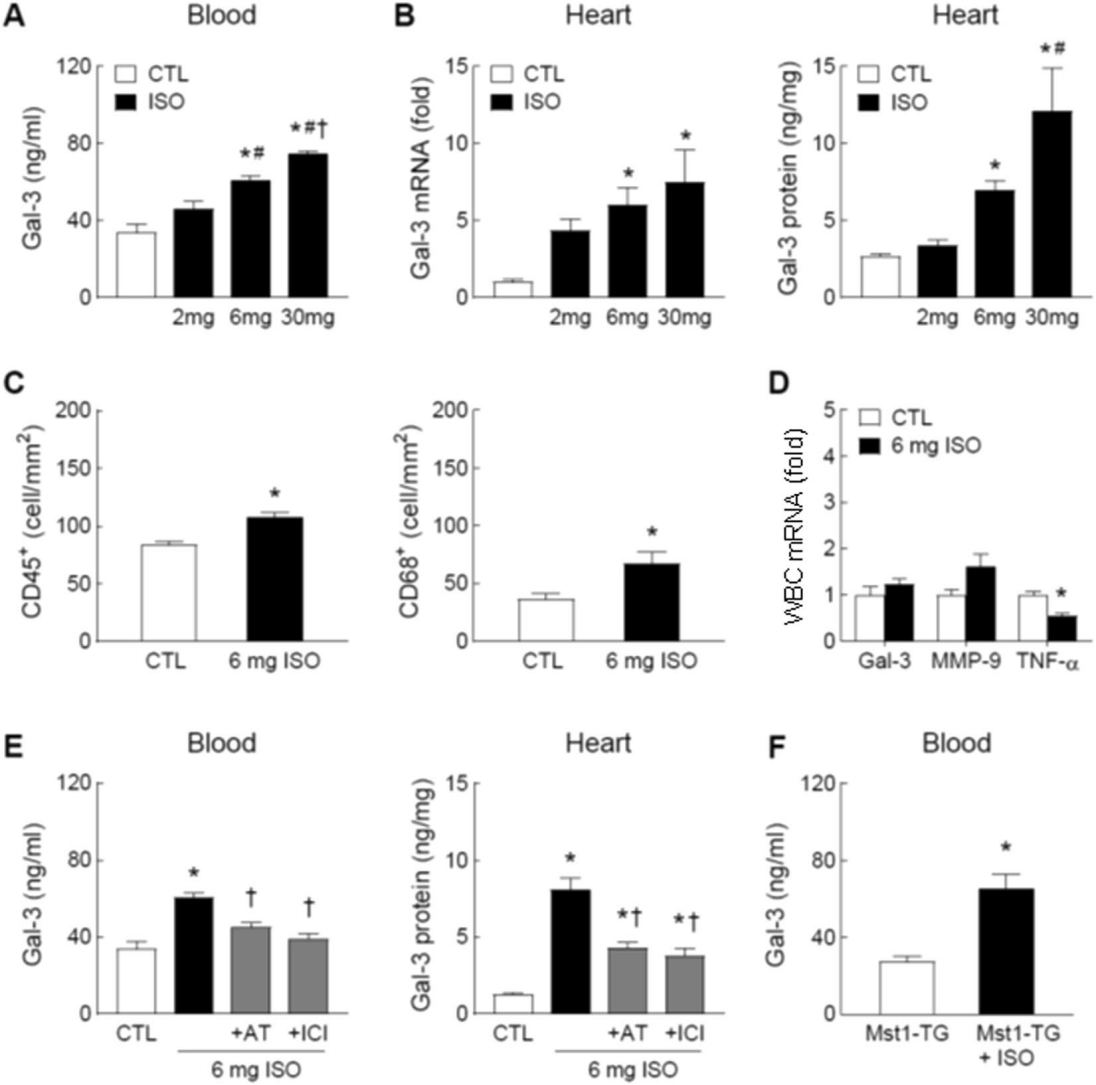
Effect of the β-adrenoceptor agonist isoproterenol (ISO) on circulating and cardiac levels of galectin-3 and inflammatory parameters. Plasma levels of Gal-3 (**A**) and cardiac expression of Gal-3 (**B**) at the mRNA and protein levels in C57Bl/6 mice treated with the β-agonist ISO for 48 h at 2, 6 or 30 mg/kg/day (via subcutaneous osmotic minipump) or control without treatment (CTL). (**C**) Density of leukocytes (CD45^+^) or macrophages (CD68^+^) quantified by immunohistochemistry in hearts of mice without and with treatment of ISO at 6 mg/kg/day for 48 h. (**D**) Expression of Gal-3, matrix metalloproteinase-9 (MMP-9) and tumour necrosis factor-α (TNF-α) genes in circulating leukocytes from mice without and with ISO treatment (6 mg/kg/day for 48 hours). (**E**) ISO (6 mg/kg/day) stimulated Gal-3 upregulation was partially inhibited by atenolol (AT, β_1_-antagonist, 2 mg/kg/day) or by ICI-118551 (ICI, β_2_-antagonist, 1 mg/kg/day), both *P* < 0.05 vs. ISO alone. (**F**) Effect of treatment with ISO (6 mg/kg/day for 48 hours) on circulating level of Gal-3 in Mst1-TG mice. *P < 0.05 vs. control (CTL) or untreated Mst1-TG, ^#^*P* < 0.05 vs. 2 mg/kg/day ISO group, and ^†^*P* < 0.05 vs. 6 mg/kg/day ISO group. Mean ± SEM. N = 5∼8/group in all experiments.

ISO treatment was used to simulate an enhanced sympatho-β-adrenergic activity in the setting of heart disease. In C57Bl/6 mice, treatment with ISO for 48 h increased plasma Gal-3 levels in a dose-dependent fashion (Fig. 5A) without change in cystatin-C levels (Fig. 2D). Cardiac expression of Gal-3 also dose-dependent increased at mRNA and protein levels (Fig. 5B). In the hearts of mice treated with ISO at 6 mg/kg/day for 48 h, densities of CD45^+^ and CD68^+^ cells were modestly but significantly increased relative to control (*P* < 0.05, Fig. 5C). Gene expression of Gal-3 and MMP-9 in circulating leukocytes was unaffected by ISO treatment whilst TNF-α was significantly reduced (Fig. 5D). Concomitant treatment with ISO (6 mg/kg/day) and either β_1_-AR antagonist atenolol (2 mg/kg/day) or β_2_-AR antagonist ICI-118551 (1 mg/kg/day) partially and significantly suppressed ISO-induced Gal-3 increase in the heart and plasma (*P* < 0.05 vs. 6 mg ISO, Fig. 5E).

To test ISO treatment as stimulus is able to increase circulating Gal-3 levels in the Mst1-TG cardiomyopathy model, Mst1-TG mice were treated with ISO (6 mg/kg/day for 48 h). ISO treatment led to a significant increase in circulating Gal-3 levels over untreated Mst1-TG mice (Fig. 5F).

### Analysis of circulating Gal-3 gradients in mice

**Figure 6:**
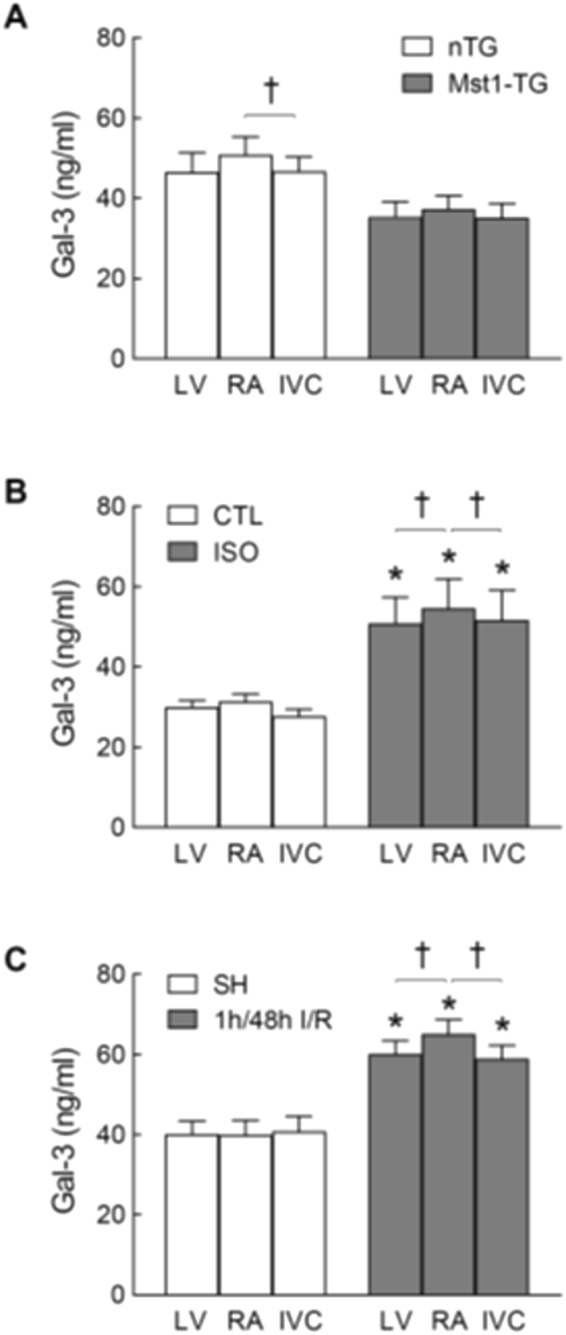
Galectin-3 gradients of plasma samples from three sites of the heart in different mouse models. In each mouse, blood was sampled from the right atrium (RA), left ventricle (LV) and inferior vena cava (IVC), respectively. Blood was collected from Mst1-TG and nTG mice (n = 9 and 6, respectively; **A**) C57Bl/6 mice treated with isoproterenol (ISO, 6 mg/kg/day for 48 h, n = 9) untreated control (CTL, n = 6, **B**) or subjected to ischemia/reperfusion (I/R, 1/48 h, n = 8) or sham-operation (SH, n = 5, **C**). Mean ± SEM. *P < 0.05 vs. CTL, SH or nTG, respectively, by two-way ANOVA. ^†^*P* < 0.05 by paired t-test.

Differences in Gal-3 levels of blood sampled from the RA, LV and IVC was determined in Mst1-TG mice and mice treated with ISO (6 mg/kg/day for 48 h) or subjected to I/R (1/48 h). Respective control mice were similarly studied. There was no Gal-3 gradient detected among samples collected from control groups, except for a significant RA-IVC gradient in nTG mice of Mst1-TG colony (Fig. 6A). In Mst1-TG mice, there was no significant Gal-3 gradient (*P* = NS by paired t-test, Fig. 6A). Mice with ISO treatment or with I/R showed higher Gal-3 levels compared to respective controls (*P* < 0.05 by two-way ANOVA, Fig. 6B,C), and Gal-3 concentration was higher in the RA than that of IVC or LV samples (*P* < 0.05 by paired t-test), implying Gal-3 release from the heart into the circulation. Gal-3 gradient was not observed in their respectively control groups (Fig. 6B,C).

### Blood levels of Gal-3 and trans-organ gradients in HF patients

**Figure 7:**
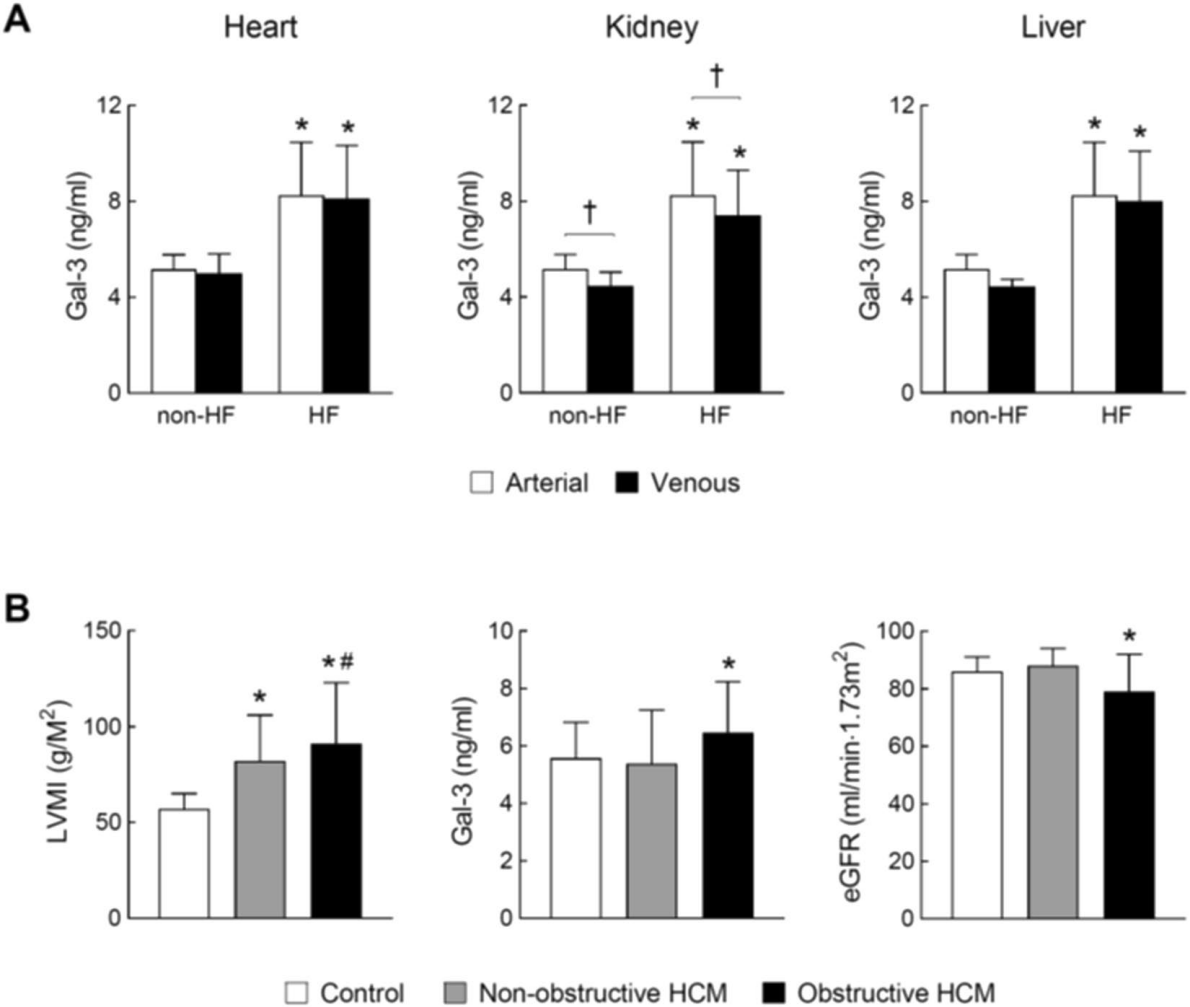
Plasma levels of galectin-3 in cardiomyopathy patients with and without heart failure. (**A**) Arterial and venous plasma levels of Gal-3 across the heart, kidney and liver from cardiomyopathy patients with HF (n = 15) and non-HF subjects (n = 5). Arterial blood was collected from the radial or brachial artery. Venous blood across the heart, kidney and liver was collected from the coronary sinus, renal vein and hepatic vein, respectively. Paired t-test was used to determine trans-organ effect of plasma Gal-3. Mean ± SD. *P < 0.05 vs. non-HF control. ^†^*P* < 0.05 by paired-t-test. (**B**) Left ventricular mass index (LVMI), plasma level of Gal-3 and estimated glomerular filtration rate (eGFR) in healthy controls (n = 20) and patients with non-obstructive (n = 27) or obstructive (n = 21) hypertrophic cardiomyopathy (HCM). Mean ± SD. *P < 0.05 vs. control group and ^#^*P* < 0.05 vs. non-obstructive HCM group.

Table 1 shows basic clinical data of cardiomyopathy patients and their respective control subjects. In HF patients due to DCM or ICM, eGFR was 51 ± 5 ml/min/m^2^, indicating impaired renal function. These patients were assessed and catheterised as a part of heart transplant evaluation and hence they all had severe HF (NYHA III∼IV) with poor LV ejection fraction (LVEF, Table 1). Compared to non-HF subjects, plasma Gal-3 concentrations were consistently higher in DCM/ICM patients with HF, irrespective of the site of blood sampling (*P* < 0.05 vs. non-HF, Fig. 7A). Trans-cardiac or trans-hepatic gradient of Gal-3 concentration was not observed. However, a significant trans-renal reduction in Gal-3 level was evident in both HF and control groups, indicating renal clearance of circulating Gal-3 (*P* < *0.05* by paired t-test, Fig. 7A).

**Table 1:** Basic characteristics of patient cohorts and controls.

	HCM	Cohort	Control	DCM/ICM	Conhort
	Non-obstructive	Obstructive		DCM/ICM	Control
Number	27	21	20	15	5
Age (year)	45 ± 14	53 ± 13	46 ± 13	56 ± 3	46 ± 10
Male (%)	74	81	70	75	80
NYHF class	I∼II	I∼II	—	III∼IV	—
LVEF (%)	67 ± 8*	72 ± 5*^†^	61 ± 6	25 ± 2	—
β-blocker use (%)	44	67	—	73	—

HCM: hypertrophic cardiomyopathy; DCM: dilated cardiomyopathy; ICM: ischemic cardiomyopathy. LVEF: left ventricular ejection fraction. *P < 0.05 vs. control, ^†^*P* < 0.05 vs^.^ non-obstrutive group.

### Circulating Gal-3 levels in HCM patients

Basic clinical data of HCM patients (n = 48) and healthy controls (n = 20) were shown in Table 1. HCM patients had disease duration of 4.5 ± 5.7 years and 25% had a family history of HCM. HCM patients were divided into non-obstructive (n = 27) and obstructive (n = 21) groups. LV mass index (LVMI) was increased by 35% and 55%, respectively, relative to healthy controls (Fig. 7B). LVEF was typically higher in HCM patients compared with control subjects, particularly in obstructive HCM group (Table 1). Plasma levels of Gal-3 were unchanged in the non-obstructive HCM group. Patients with obstructive HCM had a 20% increase in Gal-3 level together with a significant reduction in eGFR (Fig. 7B).

## Discussion

In this joint pre-clinical and clinical study, several major findings have been made regarding the factors responsible for increased circulating Gal-3 levels. *First*, elevated cardiac content of Gal-3 is a common phenomenon of heart disease whilst increase in circulating level of Gal-3 is inconsistent depending on the aetiology of cardiomyopathy. *Second*, cardiac and systemic inflammation, measured by inflammatory infiltration and upregulated Gal-3 expression in circulating leukocytes, was associated with elevated circulating Gal-3 levels. In these models, cardiac release of Gal-3 was indicated by parallel changes in cardiac and blood Gal-3 levels and by the presence of Gal-3 gradient. *Third*, activation of β-ARs leads to increased cardiac and circulating levels of Gal-3 in healthy or cardiomyopathy hearts. *Finally*, in obstructive HCM or DCM/ICM patients, the increased Gal-3 level was associated with reduction in eGFR, whilst the trans-cardiac Gal-3 gradient was no evidence in cardiomyopathy patients with severe HF.

**Figure 8:**
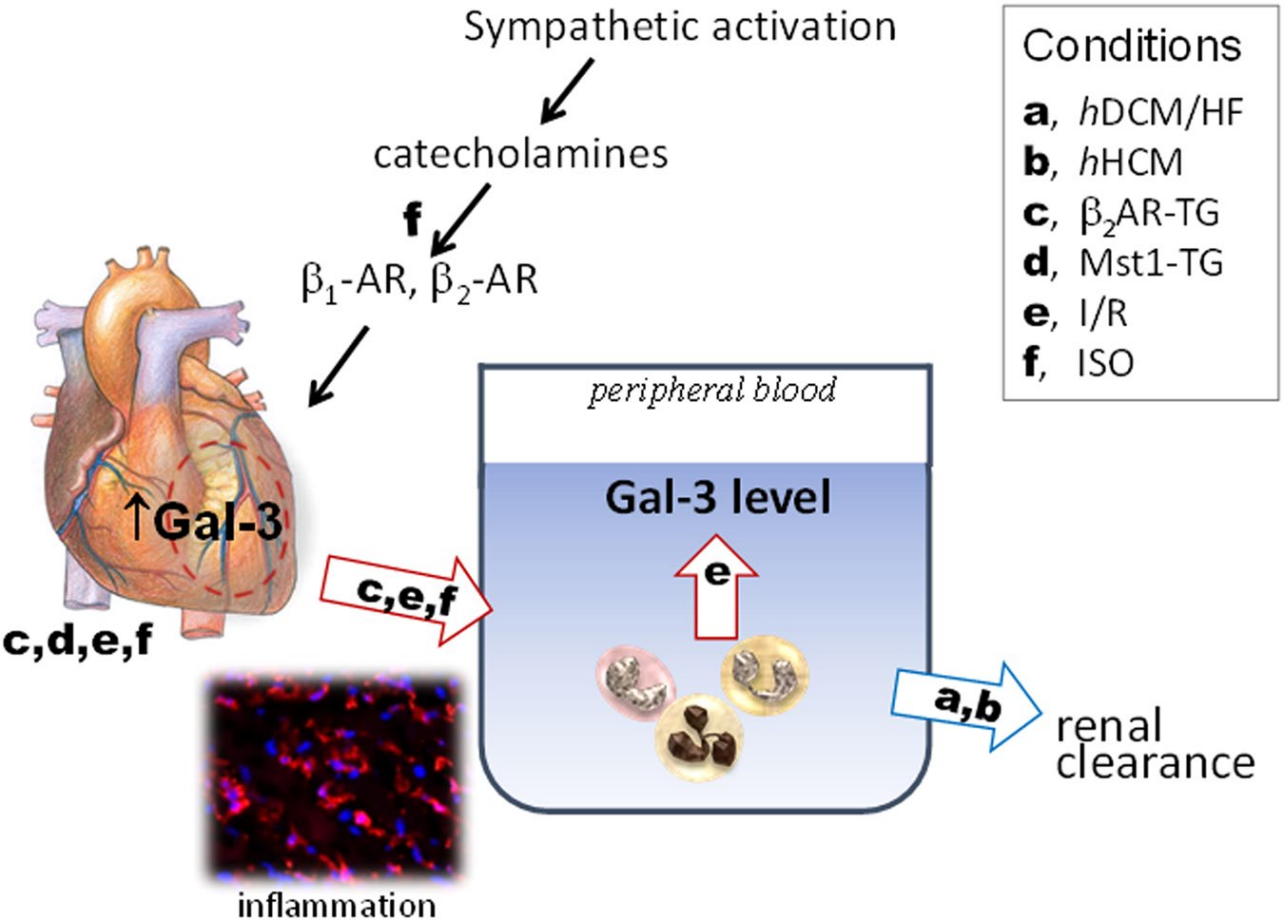
Determinants of elevated circulating levels of galectin-3 in cardiomyopathy and heart disease. Changes in circulating Gal-3 level is dependent on the aetiology of cardiomyopathy, inflammatory status and renal function. While cardiac release of Gal-3 is not observed in Mst1-TG DCM mice and HF patients (hDCM for human DCM, hHCM for human HCM), this mechanism is strongly indicated by findings from animal models of β_2_-TG cardiomyopathy, ischemia/reperfusion (I/R) or treatment with isoproterenol (ISO). Two factors closely associated with heart disease, i.e. cardiac and/or systemic inflammation and activation of the sympatho-β-adrenergic system, contribute cardiac Gal-3 release and increased blood levels of Gal-3. Red arrows indicate potential sources whilst blue arrow denotes renal clearance of circulating Gal-3 pool.

Elevation of circulating Gal-3 levels in patients with heart disease has been well documented, but an increase in cardiac Gal-3 level, relative to appropriate controls, has been rarely studied, except one report showing a significant increase in cardiac Gal-3 expression in patients with hypertensive heart disease^20^. In contrast, increased cardiac expression of Gal-3 has been consistently observed by a number of animal studies^29–34^, and yet blood levels of Gal-3 were not determined in these animal studies. Here, we reported multi-fold increases in cardiac Gal-3 expression at mRNA and protein levels in all four experimental models, which permits the examination of parallel changes of cardiac and circulating levels of Gal-3 as well as cardiac release of Gal-3 (Fig. 8). In the models of I/R, β_2_-TG cardiomyopathy or ISO-treatment, increased plasma Gal-3 levels were in parallel to that of cardiac Gal-3 expression. Furthermore, treatment with selective β-antagonists simultaneously suppressed cardiac and circulating levels of Gal-3 in ISO-treated mice. In sharp contrast, the Mst1-TG mice showed no change in circulating levels of Gal-3 nor Gal-3 gradient, albeit cardiac content of Gal-3 is the highest among the models studied. The reason for this dissociation remains unclear but does indicate that changes in circulating Gal-3 levels are aetiology-dependent. To replicate clinical conditions of stress or acute decompensation of heart disease, in Mst1-TG mice without change in blood level of Gal-3, treatment with ISO significantly increased circulating Gal-3. This finding further highlights the significance of β-AR stimulation as a key factor mediating increment of circulating Gal-3.

Absence of a trans-cardiac Gal-3 gradient was reported by two clinical studies in patients with atrial fibrillation^18^ or with hypertensive heart disease^20^. We observed in patients with advanced HF due to DCM/ICM or in Mst1-TG mice with DCM no sign of Gal-3 release from the heart. However, our findings from other animal models strongly indicate cardiac Gal-3 release as a mechanism for increased circulating Gal-3 levels. In mouse models of β_2_-TG cardiomyopathy, I/R and ISO treatment, there are parallel changes in cardiac and blood Gla-3 levels. Furthermore, mice with ISO treatment or I/R exhibited significantly higher circulating Gal-3 levels in the RA relative to LV or IVC sample, indicating cardiac release of Gal-3 into the circulation. Due to technical difficulty in sampling from the coronary sinus in the mouse, we elected to collect blood from the RA instead. It is likely that by this method, the trans-cardiac gradient of Gal-3 concentration is underestimated due to dilution of coronary sinus blood by systemic venous blood. The I/R and ISO models represent acute cardiac damage, inflammation and/or β-AR activation. Interestingly, ISO treatment to Mst1-TG cardiomyopathy mice increased circulating Gal-3. Thus it is worthwhile to test whether trans-cardiac Gal-3 gradient exists in patients in acute HF or acutely decompensated HF, acute MI or other inflammatory heart disease.

The significance of inflammation in heart disease and HF has been well appreciated by clinical findings of higher levels of inflammatory biomarkers^25^ or cardiac inflammatory cell infiltration in inflammatory cardiomyopathy, myocarditis, acute MI or I/R^16,25,26,35–38^. In the present study, we observed in the β_2_-AR TG model an age-dependent increase in cardiac density of CD45^+^ and CD68^+^ cells. Interestingly, Gal-3 levels in the LV and blood were also age-dependently increased in this model. In sharp contrast, inflammatory cell infiltration was only marginally increased in Mst1-TG hearts and yet cardiac Gal-3 level was extremely high. There are multiple cellular sources of the overall cardiac expression of Gal-3. In addition to macrophages commonly regarded as the major cell type expressing Gal-3^16,34,39^, other cardiac cells such as cardiomyocytes and fibroblasts also express Gal-3 in diseased settings^31,34,40^. In both TG models, transgene expression is restricted to cardiomyocytes and hence the high cardiac expression of Gal-3 in both models might be largely attributable to cardiomyocytes. Thus, coexistence of elevated cardiac Gal-3 expression and inflammatory status is a prerequisite for an increase in circulating Gal-3 level.

We previously showed in mice and human patients that acute MI not only leads to cardiac but also systemic inflammation^26,36^. Following MI, circulating leukocytes are activated with enhanced expression of inflammatory molecules such as MMP-9 and interleukins^26,36^. We therefore speculated that another mechanism for increased circulating Gal-3 is systemic inflammation, where activated circulating leukocytes synthesise and release Gal-3 directly into the circulation (Fig. 8). This was tested by measuring gene expression of Gal-3 as well as MMP-9 and TNFα of circulating leukocytes in all four mouse models, with results indicating that only circulating leukocytes from I/R mice showed enhanced expression of all three genes. Known as a pro-inflammatory factor, Gal-3 upregulates the expression of inflammatory cytokines and activates macrophages, promoting regional and systemic inflammation commonly seen in settings of heart disease^25^. Unexpectedly, expression of the three genes in circulating leukocytes was reduced in β_2_-AR TG model. The reason for such suppressed expression remains unclear but this rules out the presence of a systemic inflammation in this model.

Activation of the sympatho-β-adrenergic system is a hallmark of heart disease and HF^28^. Following the findings of increased cardiac and circulating Gal-3 content in the β_2_-TG model, we studied the effect of the β-agonist ISO and observed a dose-dependent elevation of cardiac as well as circulating levels of Gal-3 by ISO treatment for 48 hours. Further, use of selective β-blockers lowered both cardiac and plasma levels of Gal-3, indicating involvement of both β_1_- and β_2_-AR activation in the changes of Gal-3 observed. Considering that Gal-3 is regarded as a clinical biomarker of heart disease and HF, future studies are warranted to explore the influence of β-blockers on dynamic changes of Gal-3 circulating levels in relation to therapeutic efficacy. Whereas high doses of ISO are known to induce cardiotoxicity including inflammatory infiltration, we reported a significant but moderate increase in inflammatory cells in ISO-treated mice (6 mg/kg/day for 48 hours). Thus, elevation of cardiac expression of Gal-3 is likely attributable to cardiac cells that are equipped in high density with β_1_-AR (e.g. cardiomyocytes) and β_2_-AR (e.g. fibroblasts, cardiomyocytes).

Recent studies have well established the renal mechanism responsible for elevated circulating level of Gal-3 by showing that Gal-3 concentrations are negatively correlated with eGFR but positively with renal biomarkers like cystatin-C^13,21,23^. In the present study, all four animal models had plasma levels of cystatin-C comparable to control levels. This excluded the renal factor in the interpretation of changes in blood Gal-3 levels. This is also indicated by our finding that Gal-3 levels only increased in DCM/ICM patients or obstructive HCM patients who exhibited renal dysfunction. Whereas a negative trans-renal Gal-3 gradient was noticed in HF patients, it is likely that insufficient renal clearance explains increased plasma Gal-3 levels in these patients. Our group size of HF patients and healthy subjects was too small to identify differences in renal clearance relative to the level of Gal-3 increment.

One limitation of the present study is that we did not obtain myocardial biopsies to measure cardiac Gal-3 expression in our patient cohorts. Nevertheless, Lopez *et al*. reported in patients with hypertensive heart disease, upregulation of Gal-3 at gene and protein levels endomyocardial biopsies^20^. Another limitation is that our clinical investigation did not include conditions associated with significant cardiac inflammation. Future studies should validate our pre-clinical findings in human patients with increased cardiac inflammatory cell infiltration (i.e. I/R or myocarditis). Finally, our patient cohorts were too small to identify the influence of the use of β-blockers on Gal-3 levels, and the presence of renal dysfunction as a confounding factor makes this attempt more difficult.

In conclusion, increase in circulating levels of Gal-3 in cardiomyopathy is aetiology-dependent and mediated by different mechanisms. In cardiomyopathy and HF patients, reduced renal clearance contributes to increased Gal-3 levels without evidence of cardiac release of Gal-3. However, findings from animal models (β_2_-TG cardiomyopathy, I/R or ISO treatment) demonstrate cardiac release of Gal-3 into the circulation as a key mechanism responsible for elevated circulating Gal-3 and highlight the significance of cardiac inflammation and enhanced β-AR stimulation in promoting cardiac Gal-3 release (Fig. 8). Our findings bear translational implications for interpretation of Gal-3 as a biomarker of heart disease and potential influence of Gal-3 levels by therapies like β-blockers.

## Materials and Methods

### Animal models of heart disease

All experimental procedures were approved by the Alfred Medical Research and Education Precinct’s Animal Ethics Committee and complied with the National Health and Medical Research Council of Australia Code for the Care and Use of Animals for Scientific Purposes (8^th^ edition) and the ARRIVE guidelines. For surgical procedure or blood/tissue collection followed by euthanasia, mice were fully anaesthetized using the mixture of ketamine/xylazine/atropine (10/100/2 mg/kg, respectively).

All strains of mice were on the same C57Bl/6 genetic background. Male mice were used. Two transgenic (TG) mouse models of cardiomyopathy were due to cardiac-specific overexpression of mammalian sterile 20-like kinase-1 (Mst1-TG)^41^ or human β_2_-adrenoceptor (β_2_-TG)^42^. Our previous studies have shown phenotypes of DCM in the Mst1-TG model^41,43^ or an age-dependent development of fibrotic cardiomyopathy in β_2_-TG mice^42,44^. Age-matched non-TG (nTG) littermates were similarly studied.

I/R was induced in C57Bl/6 mice (aged 12∼14 weeks) by open-chest surgery for coronary artery occlusion-reperfusion (1 h ischemia followed by reperfusion) or sham-operated (SH), as previously described^35^. Isoproterenol (ISO, Sigma, USA) was administered to male 12∼14 week-old C57Bl/6 mice via subcutaneously implanted osmotic minipump (ALZET, USA). ISO was dissolved in saline containing 0.4 mM ascorbic acid and the doses were 2, 6 and 30 mg/kg/day, respectively for 2 days. In some experiments, animals received a combined treatment of ISO and selective β-AR antagonists atenolol (2 mg/kg/day, Sigma) or ICI-118551 (1 mg/kg/day, Sigma).

### Sampling of heart and blood and isolation of circulating leukocytes from mice

Animals were anesthetised and blood was collected by left ventricular (LV) puncture using a 25 G needle. Blood was centrifuged and plasma was collected. Hearts were harvested and the LV was separated and used for biochemical assays or immunohistochemistry. In mice with I/R, areas of the ischemic and non-ischemic LV were identified and separated with the aid of a surgical microscope.

To determine the trans-cardiac gradient of circulating Gal-3, anesthetised animal was ventilated and the chest opened. Blood was then sampled (0.25 ml per site) by needle puncture from the right atrium (RA), LV and inferior vena cava (IVC), respectively, for determination of plasma concentrations of Gal-3.

Blood leukocytes were isolated from whole blood collected in ethylene-diamine-tetra-acetate (EDTA)-coated 10 ml centrifuge tubes. Blood was diluted 1:2 with PBS/2% FBS and centrifuged at 200 g for 10 min. Leukocytes forming a concentrated ‘buffy coat’ band were carefully collected and lysed with 1x red blood cell lysis buffer. Lysed samples were centrifuged at 800 *g* for 5 min and the supernatant was removed. RNA was then extracted from isolated leukocytes for RT-PCR.

### Biochemical assays of mouse heart and blood samples

RNA from LV tissue or isolated leukocytes was extracted using Trizol® Reagent (Sigma) as previously described^44^. RNA (1 μg) was reverse-transcribed into cDNA and quantitative RT-PCR was performed using SYBR green reactions (Roche, Germany) and target primers (Sigma). The gene expression of Gal-3, matrix metalloproteinase-9 (MMP-9) and tumour necrosis factor-α (TNF-α) was measured, normalised to that of glyceraldehyle 3-phosphate dehydrogenase (GAPDH) or TATA-Box binding protein-associated factor 8 using the method of 2^−ΔΔct^, and presented relative to the control value.

Plasma levels of Gal-3 or cystatin-C (renal biomarker) were determined by ELISA (R&D Systems, USA). To measure cardiac content of gal-3 by using ELISA, LV tissues were homogenised using a lysis buffer with the addition of phenylmethylsulfonyl fluoride (1 mM), proteinase inhibitor cocktail (Sigma) and phosphatase inhibitor cocktail (Sigma).

### Immunohistochemistry

Frozen-fixed LV sections (5 μm) were incubated with primary rat monoclonal antibodies against mouse CD45 (1:50, BD Pharmingen, USA) or mouse CD68 (1:200, AbD Serotec, USA), followed by incubation with secondary antibody Alexa Fluor 546-conjugated goat anti-rat IgG (1:500, Invitrogen, USA), as previously described^35^. Nuclei were stained with ProLong® Gold antifade reagent with 4′, 6-diamidino-2-phenylindole (Invitrogen). LV sections (8∼10 per heart) were acquired using Olympus BX61 fluorescence microscope and AnalySIS FIVE software (Olympus, Australia) at x20 magnification. The number of inflammatory cells was manually quantified, in a blinded fashion, using ImageJ (NIH, USA) and the average value was used.

### Cohorts of patients with cardiomyopathy

Two patient cohorts were studied: advanced HF due to DCM or ICM, and patients with HCM. All patients and healthy volunteers were informed and consented to the study. The study was approved by the Alfred Hospital Research and Ethics Committee, which act in concordance with the guiding principles of the NHMRC of Australia.

### Heart failure patients with dilated or ischemic cardiomyopathy

We studied 15 HF patients (NYHF class III∼IV) undergoing evaluation for potential heart transplantation. Hemodynamically unstable patients were excluded from the study. The aetiology of HF was non-ischemic DCM in 69% of patients whilst the rest was due to ICM. Patients continued their anti-HF medications at the time of study. Five healthy volunteers recruited from the general community were similarly studied. They were asymptomatic and were not treated for any form of cardiovascular disease, renal disease or diabetes.

An arterial line was placed into the radial or brachial artery under local anaesthesia for blood sampling. An introducer sheath was placed into the right internal jugular or median cubital vein under local anaesthesia. A sampling catheter was sequentially positioned in the coronary sinus, hepatic vein and renal vein under fluoroscopic guidance. The tip of the catheter was positioned at least 2 cm proximal to the orifice of each vessel. Echocardiography was performed to determine LVEF.

### Patients with hypertrophic cardiomyopathy

HCM patients with confirmed HCM diagnosis by cardiac magnetic resonance (CMR) and non-HCM controls were studied. Patients were further divided into non-obstructive and obstructive HCM subgroups. All patients were in NYHF class I∼II. Peripheral venous blood samples were collected for assay of Gal-3 and creatinine. LV mass and LVEF were determined by CMR, as we previously described^45^.

### Biochemical assays of human blood samples

Blood samples were collected in EDTA-coated tubes and were subsequently centrifuged with plasma stored at −70 °C until assay. The plasma concentrations of Gal-3 were measured by ELISA (R&D Systems). eGFR was calculated following a plasma creatinine test.

### Statistical analyses

Animal data were presented as mean ± SEM and clinical data presented as mean ± SD unless otherwise stated. All results were analysed using Prism 7 Software or SigmaStat Software. Shapiro-Wilk’s and Levene’s tests were used to determine normality and equal variance, respectively. Statistical analyses included one- or two-way ANOVA followed by Bonferroni post-hoc test, unpaired student’s t-test (for datasets of only two groups) and paired student’s t-test (for paired data comparison). *P* < 0.05 was considered as statistically significant.

### Data availability

All data generated or analysed during this study are included in this article.
